# A mixed method study of medical oncologists’ perceived barriers and motivators to addressing long-term effects in breast cancer survivors

**DOI:** 10.1007/s10549-022-06657-6

**Published:** 2022-06-29

**Authors:** Alex J. Fauer, Patricia A. Ganz, Eden R. Brauer

**Affiliations:** 1 Division of General Internal Medicine & Health Services Research, David Geffen School of Medicine, UCLA, Los Angeles, CA USA; 2 Department of Health Policy & Management, Fielding School of Public Health, UCLA, Los Angeles, CA USA; 3 Center for Cancer Control and Prevention Research, Jonsson Comprehensive Cancer Center, UCLA, Los Angeles, CA USA; 4 School of Nursing, UCLA, 4-234 Factor Building, Box 956900, Los Angeles, CA 90095-6900 USA

**Keywords:** Cancer survivorship, Breast cancer, Long-term and late effects, Oncologists

## Abstract

**Purpose:**

The purpose of this study was to identify oncologist-reported barriers and motivators in addressing long-term effects with breast cancer survivors.

**Methods:**

This study is a secondary analysis of data from a survey of U.S. medical oncologists (*n* = 217) about breast cancer survivorship care in clinical practice. Using both closed- and open-ended questions, we asked oncologists to report barriers and motivators they perceived in addressing long-term effects with breast cancer patients. Descriptive statistics were used to summarize and rank items endorsed by oncologists in analyses of quantitative data; content analysis was used to identify salient categories of barriers and motivators in qualitative data.

**Results:**

Key barriers to managing physical long-term effects included lack of time during appointments (*n* = 128 oncologists, 59%) and perceived lack of evidence-based interventions (*n* = 89, 41%). With respect to psychosocial effects, oncologists reported lack of knowledge (*n* = 88, 40.6%) and challenges making referrals to mental health providers (*n* = 115, 53%). From the qualitative data, three distinct barrier categories emerged: “Competing priorities during brief appointments;” “Discussing long-term effects—Who? What? When?;” and “Beyond my expertise and comfort level.” Two motivator categories emerged: “I owe it to them;” and “Giving people a life worth living.”

**Conclusion:**

Oncologists’ key motivators for addressing long-term effects were focused on professional values, relationships with survivors, and their commitment to prioritizing patients' quality of life. Future efforts should leverage oncologists' professional and interpersonal motivators to enhance the delivery of survivorship care for breast cancer.

**Supplementary Information:**

The online version contains supplementary material available at 10.1007/s10549-022-06657-6.

## Background

There are more than 3.8 million breast cancer survivors in the United States (U.S.), and many patients with early-stage disease now have a life expectancy similar to non-affected peers [1]. However, the disease itself and the toxicity of curative treatments may contribute to morbidity and disability that persist after initial diagnosis, often described as late and long-term effects [2]. Late effects are complications that were not present at the end of treatment and occur many years later, such as heart failure or secondary malignancies [3]. Long-term effects include lingering symptoms and complications that may have occurred during and shortly after treatment, such as fatigue, pain, and depressive symptoms, but do not fully resolve [4, 5]. Therefore, ongoing assessment and management of physical and psychosocial effects is a fundamental element of comprehensive survivorship care.

With a primary focus on surveillance for disease recurrence, specialist-led models of survivorship care often fail to address the multi-dimensional needs of survivors [6, 7]. Prior research suggests that oncologist communication regarding potential long-term and late effects is often poor, leaving many breast cancer survivors uninformed, ill-prepared, and without effective symptom management for post-treatment issues [8, 9]. Although multiple clinical guidelines with clear recommendations for cancer survivorship care are available, uptake in routine care remains low, and strategies for successful integration are not well understood [10–15]. As the number of long-term breast cancer survivors increases and workforce shortages in oncology loom, traditional survivorship care models are increasingly recognized as unsustainable [6]. Better understanding of current barriers faced in clinical practice is needed to inform more comprehensive approaches to survivorship care [16].

This paper aims to identify medical oncologist-perceived barriers and motivators in addressing long-term effects in breast cancer survivors across the cancer care trajectory. Opportunities to enhance discussion, assessment and management related to long-term effects in breast cancer survivorship care are also summarized.

## Methods

### Design

This is a secondary analysis of survey data from the Medical Oncologist Survivorship Study (MOSS), conducted from October 2018 to April 2020 at the University of California, Los Angeles (UCLA) [17]. The purpose of this analysis was to examine oncologist-reported barriers and motivators to addressing physical and psychosocial long-term effects using a mixed-methods approach. MOSS surveyed a national sample of medical oncologists about various aspects of implementing guideline-concordant breast cancer survivorship care in clinical practice. Survey content was informed by findings from prior qualitative research with medical oncologists and breast cancer survivors [8], and available clinical guidelines on survivorship care broadly, breast cancer-specific survivorship care, and specific cancer-related symptoms [10–15, 18–20]. Eligible participants were U.S. medical oncologists who treat breast cancer patients, and were recruited using commercial and research databases from a professional society (ASCO), as described elsewhere [17]. Informed consent was obtained from all study participants. After completing the study survey, participants received a $50 gift card. The UCLA Institutional Review Board approved the study protocol.

### Measures

Primary outcomes of this analysis were (1) perceived barriers to addressing *physical* long-term effects, (2) perceived barriers to addressing *psychosocial* long-term effects, and (3) perceived motivators to addressing physical and psychosocial long-term effects in patients with breast cancer. The survey assessed each outcome using both closed-ended and open-ended questions. In the closed-ended format, participants were asked to “select all that apply” from a list of potential barriers based on findings from our qualitative research as well as review of existing literature. The open-ended questions allowed clinicians to write free-text responses to capture any barriers and motivators not adequately represented on the a priori list, as well as elaborate on these experiences in their clinical practice settings. The survey also queried participants about perceptions regarding role responsibility for ten activities of survivorship care, including screening for new primary cancers, evaluating for late and long-term effects, and managing comorbid conditions. Additionally, sociodemographic characteristics about participants and clinical settings were collected.

### Data analysis

We analyzed both the quantitative and qualitative data for each outcome and used the findings to identify key barriers and motivators to addressing long-term effects in patients with breast cancer. For the quantitative data, we calculated frequencies of items endorsed by oncologists in the closed-ended questions to rank individual barriers and motivators. We also used descriptive statistics to summarize demographic characteristics of the sample. Quantitative analyses were conducted in R studio, version 1.3.1073.

For the qualitative data, we used an inductive content analysis approach to analyze each primary outcome [20]. Free-text responses to the open-ended items were exported from REDCap for analysis, and reviewed by the research team. Next, we organized the data using a process of open coding, in which specific analytic labels were attached to textual data. Codes were further organized through grouping, collapsing, and comparison of salient themes across participants. These codes were then used to inform the development of initial categories. Through ongoing meetings throughout the analysis, the research team discussed and resolved any interpretive disagreements and further refined the categories and their meanings.

## Results

Data were available from 217 medical oncologist survey respondents. Their mean age was 46 years (SD 12.8 years), and most reported practicing in academic settings (*n* = 108, 49.8%) and receiving no formal training in survivorship care (*n* = 164, 75.6%). Additional characteristics of the sample are listed in Table 1.

**Table 1:** Oncologist characteristics (*n* = 217)

Physician characteristics	Subgroup	Number	%
Mean age (± SD), years	45.9 (± 12.8)		
Mean years in practice (± SD), years	19.3 (± 12.6)		
Gender	Female	79	36.4
	Male	107	49.3
	Prefer not to say	6	2.8
	Missing	25	11.5
Race/ethnicity	Non-Hispanic White	105	48.4
	Asian	59	27.2
	Hispanic White	8	3.7
	Black	1	0.5
	Prefer not to say	19	8.8
	Missing	25	11.5
Faculty appointment in a medical school	Yes	87	40.1
	No	107	49.3
	Missing	23	10.6
Training in survivorship care	Yes	29	13.4
	No	164	75.6
	Missing	24	11.1
Hours in direct patient care per week	< 30	62	28.6
	> 30	155	71.4
Usual monthly number of patients with new diagnosis of any cancer	1–10	45	20.7
	11–10	86	39.7
	21–30	56	25.9
	31–40	18	8.2
	41–50	12	5.5
Usual monthly number of patients with new diagnosis of breast cancer	1–5	97	44.7
	6–10	63	29
	11–20	34	15.7
	21–30	16	7.4
	> 30	7	3.2

### Perceived barriers to addressing physical long-term effects

**Fig. 1:**
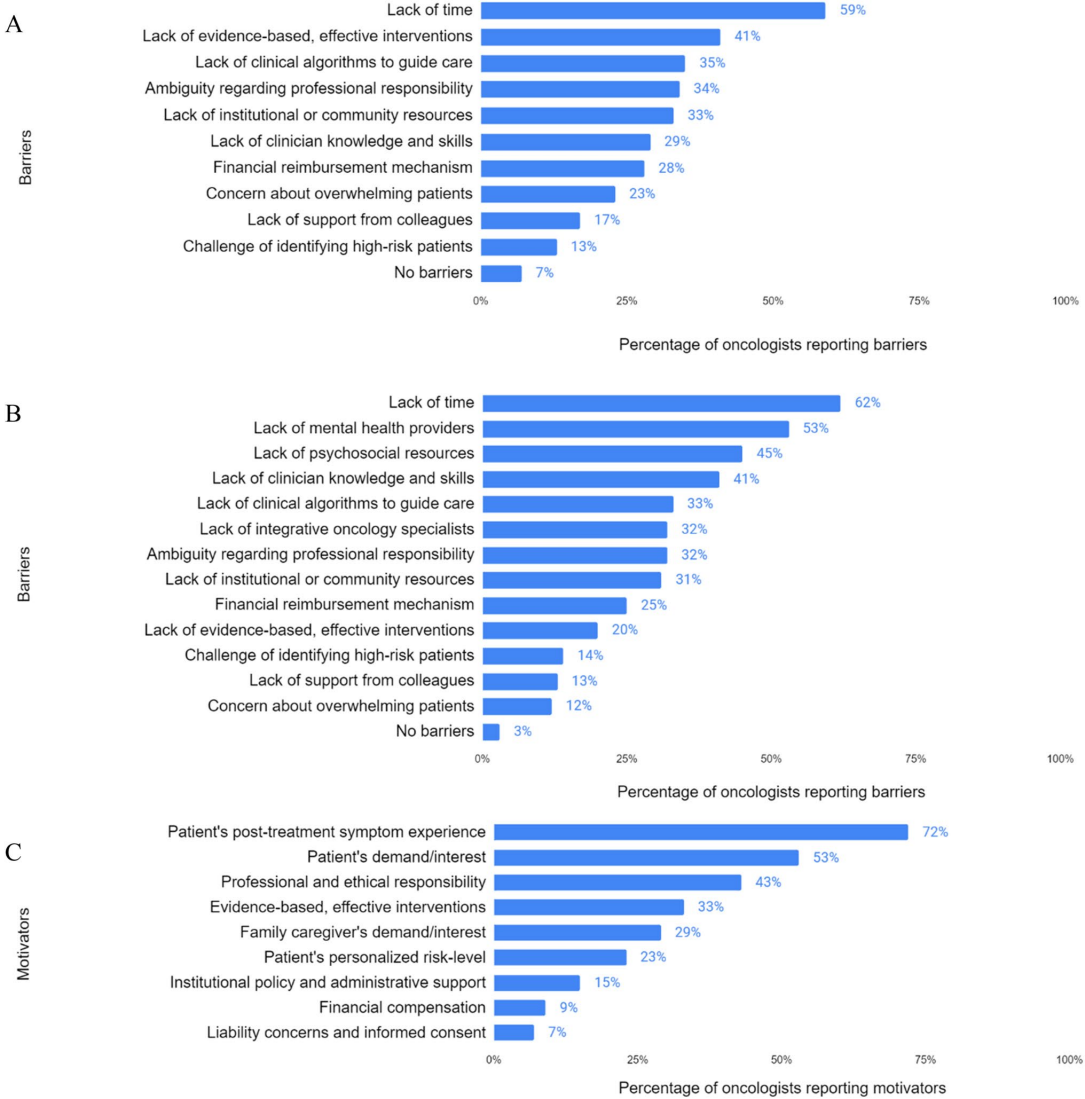
Oncologists’ perceived barriers and motivators for addressing long-term effects of breast cancer patients. **A** Barriers to addressing physical long-term effects. **B** Barriers to addressing psychosocial long-term effects. **C** Motivators for addressing long-term effects. Items are not mutually exclusive; participants were able to select all that apply

Lack of time during follow-up appointments was the most frequently endorsed barrier to addressing physical long-term effects in patients with breast cancer (*n* = 128, 59%), see Fig. 1A. Other high-ranking barriers included the perceived lack of evidence-based or effective interventions (*n* = 89, 41%) and lack of clinical algorithms (*n* = 75, 34.6%) to guide appropriate care when chronic issues are identified. Of the possible barriers, low interest among colleagues (*n* = 37, 17%) and identifying patients at high-risk for long-term effects (*n* = 29, 13.3%) were least frequently endorsed.

**Fig. 2:**
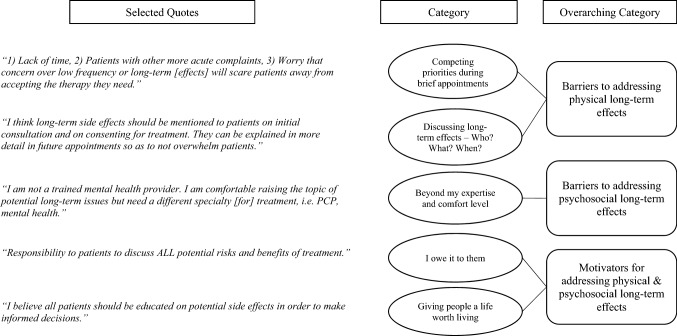
Content analysis process diagram with selected quotes from oncologists’ open-ended survey responses

In the qualitative analysis, two categories related to perceived barriers regarding physical long-term effects were identified. “Competing priorities during brief appointments” and “Discussing long-term effects—Who? What? When?” (see Fig. 2). *Competing priorities during brief appointments* refers to a tension described by oncologists between discussing long-term risks and more immediate clinical issues during pre-treatment consultations. According to several oncologists, this resulted in minimal opportunities to introduce the concept of long-term risks early in the trajectory and prepare patients for what to expect after treatment. As one oncologist shared, “There are so many [potential long-term and late effects] and of such low prevalence that higher yield of time available is spent on acute side effect preparation.” In response to time constraints, some participants expressed the desire for clinical communication tools to enhance the efficiency of these discussions during patient visits, with one oncologist suggesting, “A focused [survivorship] checklist with percentages from a national organization would facilitate the conversation.” Additional quotes representing this category are shown in Fig. 2.

The second category, “Discussing long-term effects—Who? What? When?”, reflected the complex and “overwhelming” task of initiating conversations about physical long-term effects with patients. First, oncologists expressed concerns regarding the “sheer number of possible treatment effects” for a single patient, and emphasized the challenge of consolidating and tailoring this information during brief clinical encounters. They also described ambiguity regarding optimal timing to discuss long-term effects with patients. When considering initial consultations, one participant explained, “Patients will be overwhelmed with the long-term side effects and not focus on the present.” Several oncologists shared this sentiment, with one describing attempts to avoid “overwhelming a patient before we even begin treatment.” However, others acknowledged that discussion about risks of long-term effects was necessary for patients to make truly informed decisions about treatment and thus, preferred they occur during pre-treatment consultations.

Some oncologists believed these discussions should be postponed until patients are more receptive to this information, emphasizing the end of treatment as a prime opportunity to provide anticipatory guidance and set realistic expectations with respect to long-term effects. As one oncologist explained, “Pre-treatment can be overwhelming for patients. I find a year after diagnosis, patients often just start processing their diagnosis, treatment, and plans moving forward, and we have more conversations about late or long-term treatment effects.” This lack of clarity about when to introduce potential long-term effects also contributed to the perceived difficulty of efficient assessment and management during post-treatment appointments. Additional quotes are shown in Fig. 2.

### Barriers to addressing psychosocial long-term effects

As with physical effects, the most frequently endorsed barrier to the assessment and management of psychosocial long-term effects was lack of time during follow-up appointments (*n* = 134, 61.6%). Other key barriers included the perceived lack of mental health providers (*n* = 115, 53%) and psychosocial resources (*n* = 97, 44.7%), as well as oncologists’ perception of their own knowledge and skills in this area as inadequate (*n* = 88, 40.6%), see Fig. 1B. Only seven participants (3%) reported no barriers to addressing psychosocial long-term effects in breast cancer survivors.

In the qualitative analyses, oncologists elaborated on their concerns about their own clinical abilities regarding psychosocial long-term effects, as reflected in the category “Beyond my expertise and comfort level.” This category describes how perceived gaps in their own knowledge, skills and confidence served as a barrier to addressing psychosocial needs with patients. For example, one oncologist reported, “We are not adequately trained to manage [psychosocial] issues,” while another explained that psychosocial assessment is “very personalized and hard to get at. I don’t have the training or time to get into this with all patients so I reserve it for those who I think need it.” Another oncologist reflected on “overconfidence in my own ability to manage. I do this often, but wonder if other professionals are better equipped to help my patients.” Oncologists also reported multiple challenges related to accessing psychosocial referrals and resources and closing the loop for patients who require further evaluation and support. As one oncologist shared, “Some patients have levels of anxiety that are beyond my expertise and comfort level. I have had problems referring to psychosocial services, either patients are reluctant or there no providers in their network available.”

In addition to their limited training, several oncologists identified the perceived absence of evidence-based interventions and clinical guidelines as a barrier to providing psychosocial survivorship care. As one participant shared, “I often find myself sympathizing with patients about the psychosocial long-term effects of chemotherapy, however there are few guidelines or proven therapies to address these issues.”

### Motivators for addressing physical and psychosocial long-term effects

Despite these barriers, survey respondents reported being highly motivated to try to address both physical and psychosocial long-term effects in their patients with breast cancer. When questioned about their underlying motivators, they more frequently endorsed the patients’ post-treatment symptom experience (*n* = 156, 71.9%) and the patient’s demand for or interest in post-treatment care (*n* = 116, 53.4%), see Fig. 1C. These two motivators reflect a tendency among oncologists to address long-term effects as needed, typically in response to patients’ symptom burden or patient-initiated interest. The third most frequently endorsed motivator was professional responsibility and ethical obligation (*n* = 93, 42.9%). The motivators that were reported by oncologists with the lowest frequency were concerns about financial reimbursement (*n* = 20, 9.2%) and liability (*n* = 15, 6.9%), but these were important considerations for a small subset.

In the free-text responses, oncologists described their motivation to address long-term effects across two categories, “I owe it to them” and “Giving people a life worth living.” In the first category, “I owe it to them,” participants described the ethical and professional imperatives as oncologists as key sources of motivation, using terms such as “responsibility,” “duty,” “obligation,” and “expectation.” Oncologists described this ethical obligation as a way to ensure patients are capable to “fully give consent” for treatment and clearly “understand their risks and options.” As one oncologist shared, “It is the right thing to do. If they suffer these effects, I want them to at least recall having heard it might happen.” Another shared lessons learned about communicating such issues through their own experience as a patient, “Having been through cancer treatment myself, I have some better idea of how to present issues and options (like I never got!).” Although less common, two oncologists referenced possible legal ramifications of not fulfilling these professional obligations, underscoring the need to “legally cover your bases and make sure patients fully understand what they are getting into.” Additional quotes are shown in Fig. 2.

The second category, “Giving people a life worth living,” describes oncologists’ professional commitment to quality of life, not just survival, as a main motivator in addressing long-term effects in patients with breast cancer. Many oncologists considered a focus on quality of life as a means of providing more holistic care. For example, one participant explained, “I don’t treat cancer. I treat people with cancer. I do not consider [overall survival] as the primary goal. I consider giving people a life worth living as the primary goal.” Several oncologists also believed that increasing patients’ awareness of potential long-term effects led to earlier symptom management and improved quality of life for survivors. One oncologist described these perceived benefits, “Empowering patients with information can help them feel more in control of the things they can do to help reduce and/or mitigate symptoms.” Additionally, multiple respondents viewed communication about potential long-term effects as a way of “building trust and expectations,” which also improved quality of life for their patients. As one oncologist shared, “I believe the more they know, the better prepared they are, the more trust they have in their caregiver, and the better the outcome will be.” Overall, key motivators across these categories focused on improving patients’ quality of life, building trust with patients, and upholding their professional values.

### Perceived role responsibility and survivorship care

Participants perceived the majority of survivorship care activities to be the primary responsibility of medical oncologists, PCPs, or both (Supplemental Table). Radiation oncologists and breast surgeons were perceived as less responsible for these tasks; oncology APPs were considered involved in some aspects, but not primarily responsible. Medical oncologists perceived themselves as primarily responsible for evaluating physical (86%) and psychological (64%) long-term and late effects and managing pain (81%) and fatigue (66%), while screening for new primary cancers and counseling on smoking cessation were perceived as shared responsibilities with primary care. The management of comorbid conditions was seen as the primary responsibility of primary care (75%).

## Discussion

In this secondary analysis of data from a survey of medical oncologists about breast cancer survivorship care, we examined oncologist-perceived barriers and motivators to addressing physical and psychosocial long-term effects with their patients. As the prevalence of breast cancer survivors increases and projected workforce shortages loom [6, 21], understanding barriers and motivators to addressing long-term effects in routine care is a high priority. Many barriers identified in this study, such as limited time, lack of survivorship care training, and poor communication, have been reported consistently since the Institute of Medicine *Lost in Transition* report [22, 23]. More than 15 years since its publication, it is clear that oncologists still struggle to deliver comprehensive survivorship care without structured processes in place. Although many survivorship care guidelines are now available, and oncologists acknowledge the importance of addressing long-term effects, complex barriers persist in clinical care and many breast cancer survivors do not receive adequate support.

Our findings reflect the dominant model of survivorship care, in which oncology specialists continue to follow their patients for years to decades after initial treatments, with no transition of care to other providers or settings [6, 22]. As previously reported [17], most oncologists in this study described being the sole clinician (62%) providing follow-up care, while others collaborated with an oncology advanced practice provider (APP) in their practice (16%). Only 10% of participants reported access to a dedicated survivorship clinician (MD or NP). Instead, most oncologists continue to bear the responsibility of coordinating various aspects of survivorship care without a standardized approach. This model is not only inefficient and unsustainable, but often fails to address survivorship concerns beyond recurrence, such as quality of life, physical and psychosocial consequences of cancer and its treatment, and care coordination between oncology, supportive care specialists, and patients and families [6].

Despite oncologists’ strong motivation to provide high-quality survivorship care, integrating the assessment and management of physical and psychosocial long-term effects into brief encounters is daunting. Systematic approaches are necessary to address well-known barriers, such as lack of time and poor communication, and ensure that breast cancer survivors receive adequate support. The findings of this study underscore the need for alternative models with embedded structures to facilitate systematic screening for a broad array of survivorship issues, in combination with need-based referrals and a stepped care management approach. For example, routine monitoring of survivors using validated patient-reported outcome tools may help to identify patient needs proactively and coordinate care with behavioral and integrative oncology services using an individualized, yet efficient approach [24]. Findings from this study suggest that many clinical environments lack clear processes for referring survivors for further evaluation. In these instances, oncologists may identify patient issues, but lack time or expertise to address them. Without established systems for referral, including ‘when’ (specific criteria), ‘how’ (embedded referrals), and ‘who’ (known resources), oncologists are left to construct a unique plan for each patient at each encounter. Furthermore, our sample included both generalist and breast specialized oncologists, and it is likely that addressing a wide range of potential chronic issues is even more complex for those treating patients with diverse cancer and treatment types.

To streamline this process and help clinicians identify and respond to emerging patient concerns, formalized workflows are needed to access supportive care services. Oncologists in this study described a lack of guidelines and evidence-based interventions for psychosocial long-term effects, when in fact such guidelines exist [11, 14, 15]. Screening for psychosocial distress is now considered a standard of cancer care, and many ultra-brief, validated screening tools are available for use in brief clinical encounters [20]. By standardizing this workflow, oncologists will become more familiar with existing tools and resources and how to use them, possibly reducing the time needed. In addition, clarity regarding role responsibility for specific survivorship care tasks may facilitate a team-based approach. In some settings, incorporation of lay navigators or community health workers may enhance coordination of survivorship services. Although there is growing evidence that dedicated survivorship clinics have a positive impact on patient outcomes, such as quality of life and satisfaction, compared to routine care [25], they are likely not feasible or scalable across all settings given known workforce and financial pressures. Alternative models should also be explored [6, 26, 27].

This study also highlights distinct barriers related to communication about long-term effects at different points along the care trajectory. First, consensus about optimal timing to initiate these discussions is lacking, and many oncologists described drawbacks of introducing potential long-term effects before treatment begins. However, this information was also perceived as central to informed consent. Insufficient communication about potential long-term effects early in the trajectory added to the difficulty of caring for patients once they experienced post-treatment issues because they did not receive adequate preparation or anticipatory guidance. Ideally, the risks for potential physical and psychosocial long-term effects should be threaded across the care trajectory, first introduced at pre-treatment visits and revisited in more depth as primary treatment comes to an end. This foundation may better prepare patients for long-term effects if they arise, provide guidance on self-management strategies and when to seek care, and facilitate more efficient monitoring at post-treatment encounters. Because of the close interpersonal bonds they form with survivors [28], oncologists are poised to lead these discussions, and can use them as opportunities to endorse the involvement of other disciplines in team-based survivorship care.

Expanded educational and training opportunities offer another strategy in reducing barriers in survivorship care. New research on innovative educational programs aims to improve survivorship knowledge and skills among clinicians. Smith and colleagues [29] developed a multimedia survivorship education program, intended for primary care, to bridge survivorship education gaps in routine practice. Interestingly, the program contains specific guidance on effective communication related to managing physical and psychosocial late and long-term effects using existing guidelines and evidence-based interventions. Such programs should also be available to oncologists and other disciplines involved in survivorship care, such as mental health and rehabilitative providers. This interprofessional training may help overcome barriers identified in this study, such as optimal timing and role ambiguity. However, evidence shows that educational programs alone will not suffice; focused implementation programs with audit-and-feedback strategies may lead to more effective delivery of survivorship care [23].

Despite the study’s contributions, there are noteworthy limitations to consider. This study was a secondary analysis of previous research; we were unable to seek clarification of participants’ reported barriers and motivators. We also did not specifically test oncologists’ knowledge or self-efficacy regarding best practices related to managing long-term effects. Additionally, our sample included both generalists and breast specialists and this may have impacted perspectives on survivorship care, but analyses across these characteristics were not feasible. Stratification of barriers and motivators across patient-level factors, such as age and tumor stage groups, was also beyond the scope of this study. Lastly, the study demonstrated a rigorous strategy to collect perspectives on challenges in survivorship care delivery in a national sample, though study participants may not reflect the diversity of all medical oncologists across the U.S.

Novel models for comprehensive, evidence-based survivorship care in diverse settings are needed to overcome numerous barriers in discussing, assessing and managing long-term effects as outlined in current clinical guidelines, and achieve progress in clinical practice [6, 28]. Structured processes that incorporate brief, systematic screening and need-based referrals may streamline oncologist-led survivorship care. However, their success depends on strong communication and established referral pathways with supportive care services. With increasing attention on value and sustainability, team-based survivorship care is needed to address quality of life and the multidimensional sequelae of breast cancer and its treatment.

## Supplementary Information

Below is the link to the electronic supplementary material.

Supplementary file1 (DOCX 13 kb)

## Data Availability

Datasets analyzed during the current study are available from the corresponding author on reasonable request.

## References

[1] National Cancer Institute (2001) Cancer stat facts: female breast cancer. Surveillance, Epidemiology, and End Results Program (SEER) Cancer Statistics. https://seer.cancer.gov/statfacts/html/breast.html. Accessed 4 Nov 2021

[2] Nekhlyudov L, Campbell GB, Schmitz KH, Brooks GA, Kumar AJ, Ganz PA, Von Ah D (2022). Cancer-related impairments and functional limitations among long-term cancer survivors: gaps and opportunities for clinical practice. Cancer.

[3] Kenyon M, Mayer DK, Owens AK (2014). Late and long-term effects of breast cancer treatment and surveillance management for the general practitioner. J Obstet Gynecol Neonatal Nurs.

[4] Sheng JY, Skuli SJ, Thorner ED, Zafman N, Riley CD, Ruck JM, Smith KC (2021). Late effects in a high-risk population of breast cancer survivors. Support Care Cancer.

[5] Poortmans PM, Struikmans H, De Brouwer P, Weltens C, Fortpied C, Kirkove C, Budach V (2021). Side effects 15 years after lymph node irradiation in breast cancer: randomized EORTC trial 22922/10925. JNCI J Natl Cancer Inst.

[6] Jefford M, Howell D, Li Q, Lisy K, Maher J, Alfano CM, Rynderman M, Emery J (2022). Improved models of care for cancer survivors. Lancet.

[7] Lisy K, Langdon L, Piper A, Jefford M (2019). Identifying the most prevalent unmet needs of cancer survivors in Australia: a systematic review. Asia Pac J Clin Oncol.

[8] Brauer ER, Long EF, Melnikow J, Ravdin PM, Ganz PA (2019). Communicating risks of adjuvant chemotherapy for breast cancer: getting beyond the laundry list. J Oncol Pract.

[9] Rosenberg J, Butow PN, Shaw JM (2022). The untold story of late effects: a qualitative analysis of breast cancer survivors’ emotional responses to late effects. Support Care Cancer.

[10] Bower JE, Bak K, Berger A, Breitbart W, Escalante CP, Ganz PA, Schnipper HH (2014). Screening, assessment, and management of fatigue in adult survivors of cancer: an American Society of Clinical Oncology clinical practice guideline adaptation. J Clin Oncol.

[11] Andersen BL, DeRubeis RJ, Berman BS, Gruman J, Champion VL, Massie MJ, Holland JC (2014). Screening, assessment, and care of anxiety and depressive symptoms in adults with cancer: an American Society of Clinical Oncology guideline adaptation. J Clin Oncol.

[12] Hershman DL, Lacchetti C, Dworkin RH, Smith EML, Bleeker J, Cavaletti G, Chauhan C (2014). Prevention and management of chemotherapy-induced peripheral neuropathy in survivors of adult cancers: American Society of Clinical Oncology clinical practice guideline. J Clin Oncol.

[13] Carter J, Lacchetti C, Andersen BL, Barton DL, Bolte S, Damast S, Diefenbach MA (2018). Interventions to address sexual problems in people with cancer: American Society of Clinical Oncology clinical practice guideline adaptation of cancer care ontario guideline. J Clin Oncol.

[14] Runowicz CD, Leach CR, Henry NL, Henry KS, Mackey HT, Cowens-Alvarado RL, Cannady RS (2016). American Cancer Society/American Society of Clinical Oncology breast cancer survivorship care guideline: ACS/ASCO breast cancer survivorship guideline. CA Cancer J Clin.

[15] Denlinger CS, Sanft T, Moslehi JJ, Overholser L, Armenian S, Baker KS, Broderick G (2020). NCCN guidelines insights: survivorship, version 2.2020: featured updates to the NCCN guidelines. J Natl Compr Cancer Netw.

[16] Hull O, Niranjan SJ, Wallace AS, Williams BR, Turkman YE, Ingram SA, Williams CP (2020). Should we be talking about guidelines with patients? A qualitative analysis in metastatic breast cancer. Breast Cancer Res Treat.

[17] Brauer ER, Long EF, Petersen L, Ganz PA (2021). Current practice patterns and gaps in guideline-concordant breast cancer survivorship care. J Cancer Surviv.

[18] Gradishar WJ, Anderson BO, Balassanian R, Blair SL, Bustein HJ, Cyr A, Elias AD (2017). NCCN guidelines insights: breast cancer, version 1.2017. J Natl Compr Canc Netw.

[19] National Comprehensive Cancer Network (2021) Adult cancer pain (version 2.2021). https://www.nccn.org/professionals/physician_gls/pdf/pain.pdf. Accessed 6 Feb 2022

[20] National Comprehensive Cancer Network (2022) Distress management (version 1.2022). https://www.nccn.org/docs/default-source/patient-resources/nccn_distress_thermometer.pdf?sfvrsn=ef1df1a2_4. Accessed 6 Feb 2022

[21] Elo S, Kyngäs H (2008). The qualitative content analysis process. J Adv Nurs.

[22] Institute of Medicine (2006). From cancer patient to cancer survivor: lost in transition.

[23] Fagerlind H, Kettis Å, Glimelius B, Ring L (2013). Barriers against psychosocial communication: oncologists’ perceptions. J Clin Oncol.

[24] Hahn EE, Munoz-Plaza CE, Pounds D, Lyons LJ, Lee JS, Shen E, Hong BD (2022). Effect of a community-based medical oncology depression screening program on behavioral health referrals among patients with breast cancer: a randomized clinical trial. JAMA.

[25] Gilbert SM, Dunn RL, Wittmann D, Montgomery JS, Hollingsworth JM, Miller DC, Hollenbeck BK (2015). Quality of life and satisfaction among prostate cancer patients followed in a dedicated survivorship clinic. Cancer.

[26] Yang W, Williams JH, Hogan PF, Bruinooge SS, Rodriguez GI, Kosty MP (2014). Projected supply of and demand for oncologists and radiation oncologists through 2025: an aging, better-insured population will result in shortage. J Oncol Pract.

[27] Kirkwood MK, Hanley A, Bruinooge SS, Garrett-Mayer E, Levit LA, Schenkel C (2018). The state of oncology practice in America, 2018: results of the ASCO practice census survey. J Oncol Pract.

[28] Kantsiper M, McDonald EL, Geller G, Shockney L, Snyder C, Wolff AC (2009). Transitioning to breast cancer survivorship: perspectives of patients, cancer specialists, and primary care providers. J Gen Intern Med.

[29] Smith SM, Williams P, Kim J, Alberto J, Schapira L (2021). Health after cancer: an innovative continuing medical education course integrating cancer survivorship into primary care. Acad Med.

