# Supplementary material for: A mixed method study of medical oncologists’ perceived barriers and motivators to addressing long-term effects in breast cancer survivors
Source: Breast Cancer Res Treat. 2022 Jun 29;194(3):699–707. doi: 10.1007/s10549-022-06657-6 (PMC9287210; doi:10.1007/s10549-022-06657-6)
Supplement: Supplementary file 1 — Supplementary file1 (DOCX 13 kb) [file 10549_2022_6657_MOESM1_ESM.docx]

Supplemental table: Perceived role responsibility and survivorship care activities

| **Question (no. of responses)** | **Response, n (%)** | | | | | |
| --- | --- | --- | --- | --- | --- | --- |
|  | **Medical Oncologist** | **Oncology Advanced Practice Provider** | **Primary Care Physician** | **Radiation Oncologist or Surgeon** | **No one, Not sure** | **Other** |
| Screening for other new primary cancers (n = 213) | 93 (44) | 36 (17) | 100 (47) | 6 (3) | 2 (1) | 1 (0.5) |
| Evaluating patients for late or long-term physical effects (n = 215) | 184 (86) | 20 (9) | 6 (3) | 4 (2) | 1 (0.5) | 0 (0) |
| Evaluating patients for late or long-term psychological effects (n = 215) | 137 (64) | 29 (13) | 26 (12) | 1 (1) | 14 (7) | 8 (4) |
| Counseling on diet and physical activity (n = 214) | 118 (55) | 29 (14) | 40 (19) | 2 (1) | 16 (7) | 9 (4) |
| Counseling on smoking cessation (n = 212) | 116 (55) | 19 (9) | 67 (32) | 2 (1) | 4 (2) | 4 (2) |
| Managing co-morbid conditions (n = 213) | 47 (22) | 4 (2) | 159 (75) | 1 (1) | 0 (0) | 2 (1) |
| Managing pain related to cancer treatment (n = 214) | 174 (81) | 12 (6) | 12 (6) | 3 (1) | 1 (0.5) | 13 (6) |
| Managing depression and/or anxiety (n = 214) | 94 (44) | 10 (5) | 83 (39) | 2 (1) | 4 (2) | 22 (10) |
| Managing fatigue (n = 214) | 141 (66) | 16 (7) | 46 (21) | 4 (2) | 6 (3) | 1 (0.5) |
| Managing sexual dysfunction (n = 214) | 101 (47) | 17 (8) | 54 (25) | 1 (1) | 20 (9) | 21 (10) |

Abbreviations: No., number.
